# The emergence of entrepreneurial ecosystems by capital, habitus, and practice: A two-phase model based on Bourdieu’s approach

**DOI:** 10.3389/fpsyg.2022.987485

**Published:** 2023-01-11

**Authors:** Meiling Hong, Zhenfeng Ge, Chanti Wu

**Affiliations:** The Industrial and Business Management School, Ningbo University of Finance and Economics, Ningbo, Zhejiang, China

**Keywords:** entrepreneurial ecosystem, entrepreneurship field, economic capital, cultural capital, habitus

## Abstract

Entrepreneurial ecosystems (EEs) are identified as regions with intensive and coordinated entrepreneurship practices. However, there is less focus on the longitudinal perspective to track how an EE has taken form. In this research, to understand the emergence of an EE, we developed a two-phase model with Bourdieu’s approach and identified the contents and interaction of entrepreneurship capitals, habitus, and practices in each phase. By analysing 34 interviews of technology entrepreneurs from Shenzhen, China, we found that in the heteronomous phase, pursuing economic capital and the habitus of making quick profit results in entrepreneurship practices of copycat business; and in the autonomous phase, valuing cultural capital and the habitus of altruism result in entrepreneurship practices of innovation activity. This study offers the following implications for practitioners. First, public sectors should invest in industries with high technology affordance that can create entrepreneurship opportunities. Second, social events can transform entrepreneurship practices from distributed individual level to coordinated social construction.

## 1. Introduction

The term entrepreneurial ecosystem (EE) describes an aggregation of individual elements that benefit high-growth enterprises (Isenberg, 2011). EEs are formed by the economic, political, social, and cultural environment within the social infrastructure of mentors, networks, and other entrepreneurship support provided to entrepreneurs (Stam and Spigel, 2016; Spigel, 2017; Malecki, 2018; Mohammadi and Karimi, 2021). With an abundance of essential resources like entrepreneurship-oriented policy, finance, culture, support, human capital, and markets (Isenberg, 2011), this phenomenon at first appears rather tautological: EEs often refer to regions with intensive entrepreneurial activity, and where there is a lot of successful entrepreneurship and vibrant entrepreneurial practices, there is apparently a good EE (Stam, 2015). We need to get out of this tautology to develop further insights on EE research by identifying the dynamics of EE emergence.

First of all, we need to be clear that the distinctiveness of EE relies on the centre role of entrepreneurs. On the one hand, the external economic or cultural environment influenced the practices of entrepreneurs rather than the enterprises (Spigel, 2017; McAdam et al., 2019). On the other hand, entrepreneurs’ practices are not only adjusted to the context but also take the role of creating and developing EEs (Spigel and Harrison, 2018; Thompson et al., 2018; Molina-Ramírez and Barba-Sánchez, 2021).

In other words, we can identify how an EE emerges and takes form by understanding the entrepreneurs’ practices. Based on this idea, we introduce Bourdieu’s theory of practices. At the core of (Bourdieu, 1990) theory of embodied practice are the three closely interrelated concepts of field (a social arena in which people manoeuvre and struggle in pursuit of desirable resources), habitus (dispositions: lasting acquired schemes of perception, thought and action) and capital (the resources acquired (or not) in developing habitus; McAdam et al., 2019, p. 462). Actors in a particular field will sense high-valued capital and initialise the rules of that field as a habitus. Then, in pursuit of more capital that is valuable, they will introduce practices under the guidance of that habitus; these successful practices will, in turn, strengthen the field and its habitus.

In this research, we identify the EE as a field in Bourdieu’s terms. By nature, an EE is all about engagement in entrepreneurship (Spigel and Harrison, 2018). Therefore, from the perspective of Bourdieu’s approach, the question of how an EE emerges and takes form can be rephrased into what makes people in a specific place actively cultivate entrepreneurship practices, what types of entrepreneurship capitals are highly valued, and what kinds of entrepreneurship habitus are followed.

This study developed a theoretical framework using Bourdieu’s approach to identify the emergence of an EE. From a qualitative analysis of 34 interviews of technology entrepreneurs from Shenzhen, China, we identified the content and interaction of entrepreneurship capital, habitus, and practices as they occur in the different phases of an emerging EE. Generally, regions gradually develop forms of active EEs in two phases. The first is characterised as heteronomous EE: an entrepreneurship habitus with a high preference for acquiring economic capital and less Schumpeterian innovation. In this phase, entrepreneurs are engaged in producing and selling copycats with the primary goal of profit. The second phase is characterised as autonomous EE: an entrepreneurship habitus with a high preference for entrepreneur-peer mutual trust; in this phase, cultural capital, degrees from high-ranking universities, and overseas experiences are highly valued. That is, the dynamics of an EE are embedded in the changes and interactions of entrepreneurship capital, habitus, and practices. The entrepreneurship field in a certain context becomes autonomous, which is demonstrated by signs of valuing cultural capital more than economic capital and an entrepreneurship habitus that focuses on firm growth and building mutual trust rather than merely pursuing financial benefits.

The remainder of this paper is structured as follows. Section 2 reviews the literature on the formation of EEs and related entrepreneurship studies that consider Bourdieu’s perspective. Section 3 discusses the study’s research design and data collection, while Section 4 presents the data analysis and initial findings. The final section discusses the results and concludes by arguing for the importance of understanding the dynamics of entrepreneurship capital, habitus, and practice within ecosystems and how this reinforces the emergence of EEs.

## 2. Literature review

The entrepreneurial ecosystem has gradually become synonymous with areas where entrepreneurship is vibrant (Spigel, 2017). To explain the differential performance of regional entrepreneurship, the entrepreneurial ecosystem approach followed the core idea of industrial clusters (Marshall, 1920; Porter, 1980) and regional innovation system (Cooke, 2007). Industrial clusters increase the competitiveness of new ventures in two ways: skilled workers and knowledge spillover; while in regional innovation system, the geographic ‘stickiness’ of knowledge, networks, and workers, as well as an active participation in the innovation process through policy initiatives (Spigel and Harrison, 2018). The presence of many firms and research institutes or universities help to attract resources like well-educated labour, technical knowledge, and investment. If industrial cluster and regional innovation system concepts help to understand why some places enjoy high entrepreneurship than others, it would be reasonable to wonder the distinction of the entrepreneurial ecosystem. The uniqueness of the entrepreneurial ecosystem approach is that it focuses on the leadership from entrepreneurs (Acs et al., 2017) and trajectories of innovative high-growth ventures (Spigel and Harrison, 2018).

Early works define the entrepreneurial ecosystem as “consists of a set of individual elements—such as leadership, culture, capital markets, and open-minded customers—that combine in complex ways (Isenberg, 2011).” Similarly, Cohen (2006) identified the nine most critical essential elements of entrepreneurial ecosystems as: informal social networks, formal social networks of universities, government, professional and support services, capital resources, talent pool and large firms, and infrastructure and culture. Moreover, as well-known EEs have emerged in economically developed cities, it is assumed that a region with crucial or abundant resources is a prerequisite for forming an EE (Brown and Mason, 2017). However, the resource-based view fails to explain the phenomenon of an emerging EE in a relatively resource-scarce region. In addition, the resources are not equally accessible to every entrepreneur. For example, female, immigrant, and ethnic minority groups are often socially disconnected from the main institutions (Griffin-El and Olabisi, 2017; Neumeyer et al., 2019), which exacerbates the inequality in EEs (Packard and Bylund, 2018).

Moreover, related studies have failed to establish an inevitable relationship between resources and the formation of EEs (Sorenson, 2018). One of the most convincing examples would be the comparison between Route 128 in Boston and Silicon Valley. MIT and Harvard, located in Boston, have been major forces in science and engineering research for the past 20 years and have a longer and stronger history than Stanford and Berkeley in Silicon Valley; meanwhile, Boston has consistently had the highest population density of educated people in the United States. Venture capital also first emerged in the Boston area, decades before it emerged in the Bay Area (Silicon Valley; Hsu and Kenney, 2005), yet Boston has not outperformed Silicon Valley in terms of entrepreneurial activity. Thus, a number of persuasive cases have emerged from studies and research project conducted through a cultural lens, demonstrating that differences in beliefs, rules, and values do exist across regions (Saxenian, 1996; Feldman, 2001; Aoyama, 2009; Bathelt and Glückler, 2014; Spigel, 2017), and that these differences may affect entrepreneurial activity (Davidsson, 1995), such as graduates from specific regions being more inclined to start a business than others. For example, Barba-Sánchez et al. (2022) explored the variables that influence the entrepreneurial intention of university students in Spain. They corroborated that a high degree of environmental awareness of university student’s exerts influence in their entrepreneurial behaviour. Similarly, Molina-Ramírez and Barba-Sánchez (2021) confirmed community embeddedness is an essential element of the core business, without which company creation could not happen. Meanwhile, empirical tests conducted with data from the Global University Entrepreneurial Spirit Students’ Survey (GUESSS), a project that investigates and analyses the creation of entrepreneurship in university students in various countries and regions, implicated that the entrepreneurial environment with supportive culture is an essential factor that leads to the entrepreneurship intention and propensity of engineering university (Rippa et al., 2020). But when universities lack supportive culture towards entrepreneurship, classes, professors, and guest speakers will act as role models that will increase entrepreneurial intention (Laspita et al., 2023).

As mentioned above, culture perspectives help us understand the differences of entrepreneurial activity among different regions. However, most studies assume culture as relatively static which makes the culture perspective contribute limited insights on the emergence of an EE. In other words, we need a new approach to understand how this kind of entrepreneurship culture has been forged by the dynamic interaction between the actors and culture within. Here, we introduce Bourdieu’s theory of practice to understand the dynamics of EEs by focusing on the entrepreneurs’ practices (Roundy and Lyons, 2022). Bourdieu’s approaches have gained considerable momentum as theoretical tools to understand entrepreneurship (Sklaveniti and Steyaert, 2020). It is an effective approach to overcome the dominant dichotomies (e.g., qualitative versus quantitative, agency versus structure) that exist in the study of entrepreneurial phenomena (Tatli et al., 2014).

Bourdieu’s theory can be seen as an endeavour to explain the kinds of varied resources (capitals) that individuals draw on to enact their strategies and how their strategies are both negotiated in and shaped by certain context. Bourdieu (1984, p. 101) illustrates this relationship in the following formula: habitus * capital + field = practice. The field is the social space in which actors are instantiated. Habitus refers to the individual’s dispositions, attitudes, and worldviews but flowing out of the social experience. Capitals include economic, social, cultural, and symbolic resources and competencies differentially available to individuals (Forson et al., 2014, *p*. 62).

As Bourdieu’s theory of practice proposed, entrepreneurship can be viewed as a relational process which is situated in time and space (Tatli et al., 2014). This enables us to understand dynamics of regional entrepreneurial practices. Bourdieu’s perspective has been applied to entrepreneurship research in two approaches. First, researchers try to identity the special capital that entrepreneurs have utilized (Vershinina et al., 2011; Leitch et al., 2013; Lee and Shaw, 2016; Cansiz and Tekneci, 2018; Cederberg and Villares-Varela, 2019). For example, Cansiz and Tekneci (2018) established the measurement of capital utilised by entrepreneurs in developing countries. Their empirical test shows that among the many types of capital, cultural capital (work experience) and social capital (with three or more partners) held by female entrepreneurs show a significantly positive correlation with entrepreneurial performance. Cederberg and Villares-Varela (2019) illustrated how ethnic entrepreneurs mobilise resources in the United Kingdom and Spain. They found that the ability to accumulate one form of capital might depend on the presence of other forms (e.g., cultural capital may provide access to social capital or vice versa, or cultural and/or social capital may translate into economic capital).

**Figure 1:**
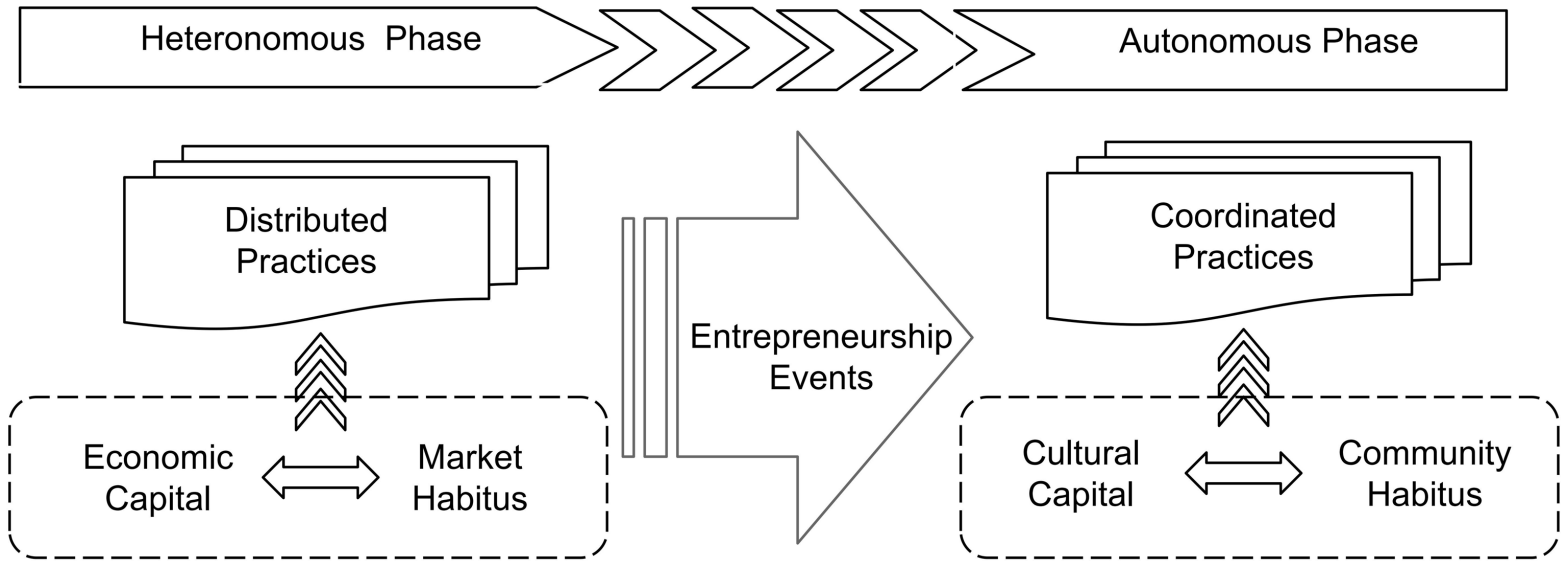
Dynamics of an entrepreneurial ecosystem: a two-phase model.

Second, recent studies strengthen the evidence that how interactions among capital, habitus, and practice influence the formation of regional EEs. For example, McAdam et al. (2019) suggested that the entrepreneurial ecosystems can be analysed in terms of Bourdieu’s theory as field, habitus, and capital. With interview data, the authors concluded that women’s entrepreneurial networks serve as gender capital for their members and improve their ability to participate in the entrepreneurial ecosystem. Accordingly, Neumeyer et al. (2019) tested whether entrepreneurs’ gender, venture type, race, ethnicity, and past venture experience influence boundaries of social capital and networks. The results suggested the habitus entrepreneurs accepted in return guide their networking style. Women entrepreneurs surpass their male counter-parts’ bridging capital scores in lifestyle and survival venture networks. Experienced women entrepreneurs that self-identified as white showed a higher degree of network connectivity and bridging social capital. Further, Opute et al. (2021) focused on how the optimisation of the dynamics in the ecosystem would drive economic growth with the incorporation of the concepts of field, habitus, and capital. Bourdieu’s theory of practice is consisting with the idea that we should emphasise the importance of regional entrepreneurship activity produced by interconnected actors (Cohen, 2006; Stam and Spigel, 2016; Turkina, 2018). Therefore, from Bourdieu’s perspective, the emergence of an EE is the expanding influence of the entrepreneurship field, whose intensive entrepreneurship practices characterise the place. A field’s autonomy is illustrated by the way it generates its values and markers of achievement (Maton, 2005). Based on the degree of autonomy, the emergence of an EE goes through two significant phases. The first phase is heterogeneous when economic capital is primarily valued. The second phase is autonomous when cultural capital is valued more highly than economic capital (see Figure 1).

Individuals bring out entrepreneurship practices based on their sense of entrepreneurship habitus and the value of the capital they hold. Thus, both entrepreneurship habitus and capitals are subject to the entrepreneurship field but with different characteristics in the two phases. The first phase is characterised as heteronomous. The entrepreneurship habitus in this phase is about acquiring economic capital with less care for Schumpeterian innovation: entrepreneurs are engaged in producing and selling products for profit only, and entrepreneurship practices are relatively distributed in the area (Thompson et al., 2018). Due to the dominant role of economic capital in the entrepreneurial landscape, entrepreneurs tend to share a common goal: to accumulate more economic capital, so that more people actively participate in a common behaviour or activity (building and expanding new businesses) and pursue a consistent outcome (positive cash flow of the business). As mentioned above, business models and entrepreneurial practices that can accumulate economic capital in the short term shape the heteronomous phase of the entrepreneurial ecosystem.

As intensive entrepreneurial practices conducted in a region, such as technology fairs and entrepreneurship competitions (Cukier et al., 2015; Thompson et al., 2018), the entrepreneurship habitus in this phase highly prefers accessing cultural capital: graduating from high-ranking universities, successful entrepreneurship experience, and high R&D investment. With the development of the entrepreneurship field, increasingly, more entrepreneurship events are held due to the successful coordination and concentration of entrepreneurship practices (Storper and Salais, 1997; Knox and Arshed, 2021). These events, like entrepreneurship competitions or startup roadshows, bring the entrepreneurs together and serve as anchors for coordinating entrepreneurship practices (Garud et al., 2014). This, in turn helps promote interactions among entrepreneur-peers and promotes the value of social and cultural capital in the entrepreneurship field. Because entrepreneurs need to establish and maintain the social relationships (e.g., colleagues and classmates) that arise from the process of acquiring cultural capital. They must adhere to community practices, helping other entrepreneurs in the community (Feld, 2012), to maintain the mutual trust within the communities because important information and resources are only shared among entrepreneurs based on mutual trust (Roundy et al., 2017). The pursuit of cultural capital and community habitus together shape the autonomous phase of the entrepreneurial ecosystem.

## 3. Case study motivation and data collection

### 3.1. Case study motivation

This study illustrates and explores the dynamics of an EE from Bourdieu’s perspective by identifying how entrepreneurial practices are developed by capital and habitus. The dynamics of an EE can be explored through a qualitative case study. Dodd et al. (2016) studied how entrepreneurs transform their capital in the entrepreneurial field, providing an example of the usefulness of this approach. Qualitative analysis allows for a nuanced understanding of how capital and habitus shape entrepreneurship practices and how the field of EEs is reshaped by practices. This work adopts the approach of Thompson et al. (2018) and Ozcan et al. (2017), using archival and interview data sources to examine the dynamics of EEs over decades in Shenzhen, China.

This approach was selected for several reasons, as elaborated. First, Shenzhen is an iconic EE in China. Home to tech giants such as Huawei, the city is one of China’s special economic zones. In the early 1980s, economic reforms introduced by Deng Xiaoping (who served as the paramount leader of China) resulted in the city becoming the first special economic zone in China, attracting overseas investment and migrants searching for opportunities. The Shenzhen government offered subsidies for banks to provide loans to startups. Thanks to this financial support, the city’s entrepreneurial, innovative, and competitive-based culture has become home to numerous small manufacturers and software companies. Because the city is a leading global technology hub, Shenzhen has been dubbed by the media as ‘China’s Silicon Valley’. In just a few decades, Shenzhen has transformed from a rural fishing village into China’s leading tech hub.

Second, we set the case in Shenzhen at the city level to avoid losing observations of relationships between variables. The case study is set at city level rather than the national level because the significance of the relationships between variables might be eliminated to a certain extent at the national level; at the same time, previous empirical research confirms that the ‘national entrepreneurship ecosystem’ itself is under suspicion (Bruns et al., 2017). The geographic boundaries of case studies should be set in cities or smaller areas.

Third, resourceful archival data have been recorded over decades; these data allow longitudinal observation and are sufficient to produce a theoretical contribution. A single case such as Shenzhen is sufficient to produce theoretical contributions (Ozcan et al., 2017). Several institutions have studied the Shenzhen EE and produced amplified reports, interviews, and archival data. We selected Shenzhen for a single case study instead of choosing several cities. Even within the same country, the level of economic development and entrepreneurial activities among cities are significantly diverse, especially in China, whose sheer size is enormous and whose social structures and financial results vary greatly (Elston and Weidinger, 2019).

Selecting the Shenzhen EE to conduct a single case study allows for examining a previously unobservable or rare instantiation of a particular phenomenon longitudinally and at a fine-grained level of detail, an approach that would not be feasible with multiple cases (Ozcan et al., 2017). Overall, the case of Shenzhen EE is sufficient to produce theoretical contributions. The representativeness of the case is not reflected in statistical sampling logic but in the variable relationship reflected in the case study or in the representativeness of the dimension being studied. Although the study examined only one area in Shenzhen, the dimensions of the research focusing on entrepreneurship capital, habitus, and practice do not exist only in Shenzhen, so the research findings are generalisable.

### 3.2. Data collection

Following Thompson et al.’s (2018) approach, we employed an inclusive sampling strategy in the focus region. We collected data from June 2015 to September 2018, drawing from semi-structured interviews, archival documents, and media sources. We utilised various data from 1999 to observe the dynamics of the Shenzhen EE. There were two sources for our data collection: semi-structured interviews and public reports and statistics. First, we conducted semi-structured interviews with entrepreneurs to identify the content of entrepreneurship capital, habitus, and practice and their dynamic interactions. The interviews of entrepreneurs primarily followed four themes: (1) education and work experience; (2) funding experience; (3) social networks; and (4) description of entrepreneurship practice patterns in their region. We transcribed 34 semi-structured interviews with Shenzhen entrepreneurs (numbered as E00X). There are four selection criteria for these entrepreneurs. First, all entrepreneurs should have registered their enterprises in Shenzhen. Second, their business scale should no more than 50 employees. Third, interviewed entrepreneurs should from information and communications technology industries. Fourth, entrepreneurs who have established their business after the year 2009 should have been based in business incubators at least once.

Of these entrepreneurs, 13 established their first enterprise between 1999 and 2008, and the remaining initially engaged in entrepreneurship between 2009 and 2018. Each interview lasted from 30 to 90 min, and the interviews were recorded, transcribed, and coded with NVivo.

The choices of how many interviews are decided by following rules. Firstly, in this research we focus on a single region: Shenzhen entrepreneurial ecosystem. As a single case study, the choice of how many interviews depend on the availability of other types of data sources. Not only we conducted semi-structured interviews but also collected archives and media materials over 40 years of Shenzhen’s experience on innovation and entrepreneurship. Secondly, in comparison with previous research on regional entrepreneurship, we observed that 34 is an acceptable number. For instance, Yamamura and Lassalle (2020) drew on their findings of different forms of proximity allow for development of EE in Malta by 10 interviews. Similarly, Lee and Shaw’s, 2016 analysed interviews of 10 entrepreneurs to identify what forms of non-physical capital are included for startups. In the study of non-linear evolution of the Vienna EE, the authors had 22 semi-structured interviews (Radinger-Peer et al., 2018). Thirdly and most importantly, we documented the interviews in with detailed notes, and we stop conducting new interviews when there were no further constructs emerged during the subsequent analysis.

Second, to study the complex process of Shenzhen’s EE over an extended period, we checked the open-source reports and statistics, including Shenzhen Annual Statistics from 1999 to 2018, Annual Report of Guangdong Province, Shenzhen Electronic Information Industry Report, policies of the Shenzhen Municipal People’s Government of Shenzhen on innovation and entrepreneurship, and related news and reports. This open-source information sorts out the policy environment, the development history of regional pillar industries, the economic environment, and overall entrepreneurship for case analysis. Efforts made to understand the entrepreneurial practice patterns shaped by capital and habitus in the development of the entrepreneurship field yielded data spanning from 1999 to 2018.

Our first observation period was from 1999 to 2008. The beginning of our first observation period, 1999, was the first time that Shenzhen’s GDP ranked fourth in mainland China, and the city subsequently maintained this ranking for years. In the meantime, economic growth was primarily boosted by the electronic manufacturing industries of Shenzhen. However, Shenzhen’s electronic manufacturing was known by a copycat symbol or ‘shanzhai’. ‘Shanzhai’ translates to ‘mountain fortress’, referring to a gang-controlled monopoly outside government control. ‘Shanzhai’ is usually used to describe the production and sale of cheap local facsimiles of globally branded goods. However, there is more to ‘shanzhai’ than mere imitation. It is considered a skill to create exact copies of original works and is part of the path towards mastering one’s craft. Not only was the city’s working-class contributing to the city’s technological development, but it was also competing with some of the biggest tech brands in a wild ecosystem model. Therefore, from 1999 to 2008, Shenzhen’s EE was labelled by copycat electronic products and Shenzhen’s famous Huaqiangbei shopping district. This 1-km stretch of technology and hardware stores is where the city’s buzzing culture of innovation is on full display. New products are created every day; makers look at products currently on the market and find ways to improve them to create entirely new pieces of technology, with the amplified opportunity to discover and pursue new business.

With the rapid development of electronic industries and copycat makers, the local government needed to encourage innovation and entrepreneurship. Thus, the first China Hi-Tech Fair (CHTF) was held in 1999, marking Shenzhen EE’s emergence. The CHTF is an international trade fair for electronics and electrical engineering, which occurs annually at the Convention and Exhibition Centre in Shenzhen. Thus, the CHTF acted as a catalyst for intensive entrepreneurship practices.

First, it provides a communication platform for entrepreneurs from electronic related industries. This kind of social and commercial communication is effective for small business performance. Moreover, business owners believe that their new product development does not come merely from research and development but also from frequent exhibition activities, which enabled knowledge and experience sharing.

Second, the CHTF promotes the commercialisation of new technologies by auctioning technological achievements and high-tech intellectual property transactions. With the popularity of the CHTF, intellectual property transactions have gradually been acknowledged in the entrepreneurship field, not just as an annual activity of the CHTF. It provides a massive boost to the commercialisation of technological achievements, transforming transactions from rare events to daily practices.

Third, the CHTF introduces financial resources to Shenzhen entrepreneurs. Twenty years ago, venture capital (VC) was scarce in mainland China. The CHTF offers a convenient channel for local entrepreneurs to seek funds from domestic and overseas institutions. The most well-known example is Pony Ma, who established the tech giant Tencent and achieved his prime venture capital from the first CHTF. Without CHTF attracting foreign investment, Shenzhen entrepreneurs would have continued struggling with few funding opportunities.

Our second observation period was from 2009 to 2019. The beginning of the second observation period, 2009, was set in the year that ChiNext was finally inaugurated. ChiNext is a NASDAQ-style subsidiary of the Shenzhen Stock Exchange. It aims to attract innovative and fast-growing enterprises, especially high-tech firms, and its standards are less stringent than those of the Main and SME Boards of the Shenzhen Stock Exchange. Although China began cultivating its VC industry in the 1980s (Ahlstrom et al., 2007), the development of domestic VC was stifled due to the lack of divestment opportunities as there was no NASDAQ-style segment in the Chinese stock market (White et al., 2005). The launch of ChiNext has strengthened Shenzhen’s importance as a VC centre to support innovation and entrepreneurship. A group of innovative enterprises successfully raised funds through the capital market. Although entrepreneurs in the early stage cannot directly benefit from ChiNext, its establishment represents an optimistic attitude from the financial market, which makes funding easier to procure. In the meantime, financial institutions have multiplied, and the public sector has launched a series of policies to encourage and regulate the development of VC institutions. The Shenzhen government established bank-government-enterprise cooperation to provide lower interest rate loans to small businesses. WeBank, which was established in 2004, is the first privately owned digital-only bank in China focused on inclusive finance and banking. WeBank does not rely on property guarantees and grants loans through face recognition technology and big data credit ratings. The rapid growth of Shenzhen’s financial market supports local entrepreneurship practices.

In addition to funding resources, a more critical feature of this period is that various entrepreneurial competitions in Shenzhen have become regular activities. Due to the growing entrepreneurship culture in the region, Shenzhen has seen several annual entrepreneurship activities. These include the first innovation and entrepreneurship-related competition, Entrepreneurship Star Competition in 2008, the Innovation and Entrepreneurship Competition (China, Shenzhen) in 2009, and the Shenzhen–Hong Kong Youth Entrepreneurship Competition in 2011. Entrepreneurship competitions have improved society’s focus on entrepreneurs, while the increased exposure of entrepreneurial enterprises has provided entrepreneurs with access to VC. At the same time, such activities promote entrepreneurs’ thinking about their business models and corporate growth and provide more timely feedback on the effects of public sector policies and regulations.

## 4. Data analysis and findings

### 4.1. Content analysis: Identifying entrepreneurship capital

The first stage of data analysis focused on coding semi-structured interview transcripts to identify the content of the entrepreneurship capitals essential for entrepreneurs in different phases of EE. Our data show that economic, social, and cultural capitals are frequently mentioned. However, the components of each type of capital varied in different phases of EE (see Table 1). First, for example, in the heteronomous phase of EE, entrepreneurs use their savings, salaries, or funds from family and friends as economic capital; in the autonomous phase, entrepreneurs begin accessing economic capital in the form of VC or loans. Second, for social capital, in the heteronomous phase, entrepreneurs mainly rely upon ‘lao xiang’; this term refers to people who share the same geographic origins of birth or childhood. Hence, entrepreneurs often put their highest trust in their ‘lao xiang’, relatively independent of the surrounding social structure. Even strangers can build mutual trust and social connections once they find out that they share the same geographic roots, such as once living in the same village or attending the same school. Evidence found in our research data shows that entrepreneurs who started their businesses in the heteronomous phase (1999–2008) were initially based in Chaoshan, a city near Shenzhen, but they retained their unique dialects and traditions. In the autonomous phase, entrepreneurs maintained their social capital, which consisted of former colleagues or entrepreneur-peers and shared professional knowledge. Third, while for cultural capital, in the heteronomous phase, most entrepreneurs possessed and valued relative working experience in electronic industries; in the autonomous phase, overseas experience or higher education degrees become essential in order to establish a tech startup in Shenzhen.

**Table 1:** Entrepreneurship capital components from Shenzhen entrepreneurs’ interviews.

Variables	Contents	1999–2008: heteronomous EE	2009–2018: autonomous EE
Entrepreneurship capital	Economic capital	Savings	Bank loans
		Salaries	Venture capital
		F&F money	
	Cultural capital	Working experience in electronic industries	Overseas working or studying experience
			Postgraduate degree
	Social capital	‘lao xiang’* resource bonding	Former colleagues
			Entrepreneur-peers
			Resource bridging

^*^Lao xiang refers to people who share the same geographic origins by birth or during childhood.

### 4.2. Content analysis: Identifying entrepreneurship habitus

Entrepreneurship habitus refers to the specific social norms or rules widely sensed and followed by entrepreneurs to conduct their practices. Shared values among ecosystem participants are critical for creating cohesion among participants, which produces some correlation among their actions and provides structure to the system. Habitus is created through a social process that leads to enduring patterns that are transferrable from one context to another, but that also shift with specific contexts over time. From the interview transcripts, entrepreneurs follow a significantly different habitus when they start a business (see Table 2).

**Table 2:** Entrepreneurship habitus components from Shenzhen entrepreneurs’ interviews.

Variables	Contents	1999–2008: heteronomous EE	2009–2018: autonomous EE
Entrepreneurship habitus	Market	To make a quick profit	To develop innovative products and make enough profit
	Community	N/A	To build mutual trust among entrepreneurs
			To establish collaborations among entrepreneurs

Entrepreneurs who started their own business during the heteronomous phase (1999–2008) focused much more on quick profit with less attention to innovation; thus, imitative entrepreneurs are prominent. Those who engaged as entrepreneurs during the autonomous phase (2009–2018) argue that only by developing innovative products will they make enough profit, and entrepreneurs will be despised if they are involved in imitating and trademark infringement of brands and other companies. Furthermore, when limited business incubators and co-working spaces are built in the heteronomous phase, it is difficult for entrepreneurs to establish mutual trust and collaboration among peers. However, in the autonomous phase, due to business incubators and events, it is much easier for entrepreneurs to share a related set of goals and behaviours focused on cooperation and altruism among peers.

### 4.3. Longitudinal analysis (1999–2008): Entrepreneurship practices in the heteronomous phase

As mentioned above, intensive entrepreneurship practices in the region forged the EE, and these entrepreneurship practices are affected by the capital held or pursued by entrepreneurs and their perceived habitus within the entrepreneurship field. Nevertheless, the degree of autonomy in the field is relatively low initially, which means the field is highly influenced by economic capital. From 1999 to 2008, the Shenzhen entrepreneurial ecosystem was labelled by its copycat electronic products. During this time, the most valued form of capital was economic capital, and financial performance became the primary criterion for evaluating entrepreneurship success. Entrepreneurship was more effective for obtaining financial benefits than being employed. To quickly obtain economic capital, entrepreneurs often ignored being engaged in producing copycat products. Our interview data show that among the 13 interviewees who became entrepreneurs between 1999 and 2008, four were Huaqiangbei vendors who once sold copycat electronic products and later established mobile phone businesses, producing and selling repair parts or phones. The remaining nine entrepreneurs were engaged in the electronics business (see Table 3).

**Table 3:** Entrepreneurs who established enterprises between 1999 and 2008.

No.	Previous experience	Main business
E008	Experienced engineer	Personal computer assembling service
E009	Earned master’s degree from high-ranking university	Electronic equipment vendor
E010	Experienced engineer	Software development
E011	Former Huaqiangbei vendor	Mobile phone assembling service
E012	Former Huaqiangbei vendor	Mobile phone assembling and repairing service
E013	Former Huaqiangbei vendor	Mobile phone assembling and repairing service
E014	Experienced engineer	Software development
E015	Experienced engineer	Software development and chip-design service
E016	Working experience in the United States	Electronic product designing service
E017	Working experience in the United States	Personal electronics device
E018	Experienced engineer	Chip assembling service
E019	Former Huaqiangbei vendor	Mobile phone assembling service
E022	Experienced engineer	Software development for automobile industries

Interview data.

On the one hand, if one wants to establish their own business in Shenzhen, being immersed in the electronic industries and producing electronic devices that look like top brands can assure a prosperous life. An entrepreneur (E002) said, ‘It felt like zeitgeist… to start doing business in Shenzhen. Families and friends were all rushing into electronic industries, and I was ignited as well’. On the other hand, there are some innovative adjustments even in copycats. For example, mobile phones designed with traditional fortune-telling functions are popular in the local market. Although these innovations seem more like tinkering, increasingly more people are becoming Schumpeterian entrepreneurs and gradually forging Shenzhen EE. As one entrepreneur (E011) recalled, ‘At first, everyone in Huaqiangbei sold copycats… but in the end it is a price war, and everyone is trapped. You have to be innovative and brave’.

**Figure 2:**
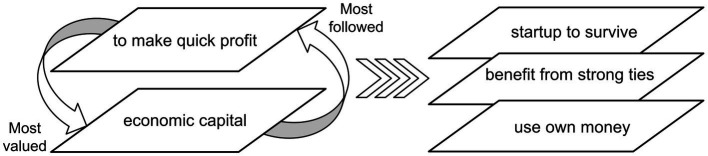
Entrepreneurship practice patterns (heteronomous phase: 1999–2008).

As above, in the heterogeneous phase of Shenzhen’s entrepreneurial ecosystem (1999–2008), the most valued form of capital was economic capital; in this context, making a quick profit was what most people sensed and followed. These factors influence entrepreneurship practice patterns (see Figure 2). First, the motivation for entrepreneurship is to make money and become rich. People who started businesses during this period did not see themselves as entrepreneurs. Even entrepreneurs who studied abroad or possessed higher degrees argue that their only motivation was to live a prosperous life. Second, regarding social activities, most people prefer to maintain their original social ties (families and close friends) and not expand their social networks to the professional domain. Third, self-funding was popular for initial funding. Entrepreneurs worried that outside investment would cause them to lose control over their businesses.

### 4.4. Longitudinal analysis (2009–2018): Entrepreneurship practices in the autonomous phase

As entrepreneurship practices become more intense, the entrepreneurship field is enhanced. Activities such as technology exhibitions and entrepreneurial competitions improve the acceptance of entrepreneurship practices. At the same time, these events promote communication and interaction among entrepreneurs. Entrepreneurs need to utilise their cultural capital to penetrate specialised networks, such as working in a famous company or learning at top universities. When they have become a member of a community, they focus on altruism. As cultural capital and the habitus of altruism play increasingly significant roles in conducting entrepreneurship practices, economic capital no longer has absolute dominance over the entrepreneurship field.

From 2009 to 2018, Shenzhen gradually escaped its copycat identity and witnessed many innovative activities. From our interview data, entrepreneurs who initially engaged in their own business from 2009 to 2018 hold undergraduate degrees from high-ranking universities either locally or abroad (see Table 4). Remarkably, nine of 21 entrepreneurs studied or worked in either the United Kingdom or the United States. They all have working experience in cutting-edge technologies. Moreover, seven out of 21 entrepreneurs are former employees of tech giants in Shenzhen, namely Tencent, Huawei, and Foxconn. Instead of economic capital, cultural capital plays the most crucial role. The habitus changed from making a quick profit to making enough profit and being altruistic. This is evident in the entrepreneurs’ interviews. For example, a software company owner who has been a PhD candidate (E029) reported that, ‘…entrepreneurs know entrepreneurs well; we have this natural emotional attachment to our peers. The communication between entrepreneur-peers often brings me effective solutions. We help each other. It is mutual trust and benefit’.

**Table 4:** Entrepreneurs who established enterprises between 2009 and 2018.

No.	Previous experience	Main business
E001	Former Huawei engineer	Cloud-computing service
E002	Working experience in the United Kingdom	AI and software development
E003	Earned master’s degree from high-ranking university	Software development
E004	Earned master’s degree from high-ranking university	Digital equipment
E005	Working experience in the United States	Laser-tech based equipment
E006	Working experience in the UK	Digital control technology
E007	Working experience in the United States	Bio-tech based product
E020	Earned doctorate in the United States	Cloud-computing service
E021	Earned doctorate in the UK	Software development
E023	Working experience in the United States	Electrical engineering service
E024	Earned doctorate in the United States	Transducer design
E025	Former Huawei engineer	Wearable smart devices
E026	Digital simulation technology researcher	Wearable smart devices
E027	Former tencent engineer	Data storage service
E028	Earned doctorate in the United States	Bio-tech product
E029	Dropped out PhD candidate	Software development
E030	Former Tencent engineer	Visual-tech service
E031	Former Tencent engineer	e-Commerce platform operation service
E032	Former Foxconn engineer	Business consulting service
E033	Earned master’s degree from high-ranking university	Digital devices
E034	Former Tencent engineer	Data storage service

Interview data.

**Figure 3:**
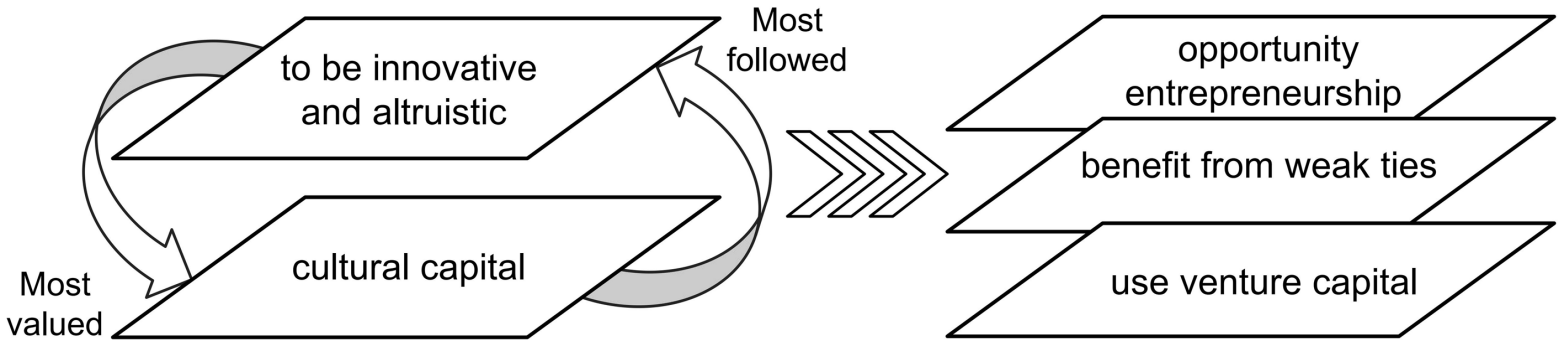
Entrepreneurship practice patterns (autonomous phase: 2009–2018).

The entrepreneurship practices in the autonomous phase distinguish it from the heteronomous phase (see Figure 3). First, regarding entrepreneurship motivation, although the primary purpose of making a good fortune has not been ruled out, people assume their identity as ‘entrepreneurs’ and want to be among the ‘tech-elite’. Second, concerning social activities, reaching out for other entrepreneurs or experts is precisely what entrepreneurs in the autonomous phase take for granted. When in need of funding, both VC and M&A are welcomed by most entrepreneurs without concern about losing control of their companies.

## 5. Discussion

This paper aims to get rid of tautology in EE research. The starting point of our research is directed by the core idea of Bourdieu’s theory of practices. The case of Shenzhen shows that an EE is not just emerging in resourceful regions. The dynamic emergence of an EE can be identified as a two-phase model, based on the degree of autonomy in the entrepreneurship field. When the field (EE) is in a lower degree of autonomy, entrepreneurs are constrained to heteronomous phase of an EE, which means the ostensibly neutral activities in the field are to make a quick profit by imitation. While in the autonomous phase, imitation is disdained, and innovation becomes the duty of entrepreneurs.

We also identify the features and content of entrepreneurship capital, habitus, and practices under EE context. Firstly, we identified a neglected manifestation of social capital, ‘*lao xiang’*, in EE research. This social capital is embedded with geographic origins of birth or childhood, which is relatively independent of the surrounding social structures (Villanueva et al., 2018; Molina-Ramírez and Barba-Sánchez, 2021; Zhou, 2022). What is surprising that, even strangers can build mutual trust quickly once they find out that they share the same geographic roots. Our finding elaborates the understanding of social capital in China context, the identification of ‘*lao xiang’* helps entrepreneurs obtain essential resources by converting weak ties (distant social relationships and infrequent interactions, which are commonly observed between acquaintances or strangers) to strong ties (close-knit members with frequent interactions; Granovetter, 1973).

Secondly, entrepreneurship habitus is created through a social process that leads to enduring patterns that are transferrable from one context to another, but that also can change over a long historical period (Navarro, 2006). At first, to effectively obtain financial benefits, entrepreneurs were not shamed of producing copycat products, but they were constantly make innovative adjustment to the products(York and Venkataraman, 2010), to live up to the duty of entrepreneurship. This phenomenon was also assumed by Keane and Zhao (2012) as echoing the ethos of contemporary hacker culture. Moreover, entrepreneurs gradually changed the entrepreneurship habitus from cooperation between families to reciprocity within enlarged entrepreneurs’ community.

Thirdly, collective and intensive practices forge the ecosystem, and the ecosystem reinforces particular practice patterns (Forson et al., 2014; Tatli et al., 2014; Dodd et al., 2016). With the local government encourage innovation and entrepreneurship (Wei, 2022), activities such as technology exhibitions and entrepreneurial competitions in Shenzhen strengthened entrepreneurship field (Cukier et al., 2015).

Beyond these theoretical contributions, our study offers practical implications for public sectors. Initially, Shenzhen was relatively scarce of entrepreneurial resources such as finance, technology, and talent. Currently, Shenzhen has shown coordinated and collective entrepreneurship. The formation and development of Shenzhen’s EE have provided some learning experience for other regions. On the one hand, it is essential for public sectors to invest in industries with high technology affordance (Autio et al., 2018). In the case of Shenzhen, the local government provides fund and tax-deduction to information and communications technology industries, which create potent digital affordances that likely have an economically transformative effect on the organisation through innovation (Nambisan, 2018). On the other hand, it is vital for practitioners to organise and participate in social events to share resources and tacit knowledge on business expertise. Although there is a contingency in the emergence of EEs, social events like entrepreneurship competition or startup salons lead to change from distributed to collective and coordinated entrepreneurship practices in the region.

However, this work has certain limitations. Due to the research design of this study, we have not worked on further comparative analysis among various EEs in developing economies. In future research, comparative analysis of EEs in multiple regions can be carried out using qualitative comparative analysis methods to study the impact of different configurations of habitus and capital on local entrepreneurship practices.

## 6. Conclusion

To depict the dynamics of an EE, we employed insights from Bourdieu’s theory of practice. In Bourdieu’s approach, we see the nature of an EE as an entrepreneurship field. Shaped by the ‘collective beliefs and values’ that emerge (Hinings et al., p. 305), entrepreneurship practices forge the local EE. Bourdieu’s approach enriches ecosystem theories by considering how individual practices contribute to the dynamics of an EE over decades.

First, intensive entrepreneurship practices are the reason an area becomes an EE, not results. Our conclusion is consistent with Spigel and Harrison’s (2018) opinion that an EE is about regional entrepreneurship engagement. The definition of who should or should not be a stakeholder of an EE is unnecessary. For instance, universities are often considered essential stakeholders of regional EEs (Civera et al., 2019; Pugh et al., 2021) because they can host entrepreneurship training programmes to encourage academic entrepreneurship and spinoffs. However, from our cases and archival data, universities in Shenzhen did not act as talent magnets or business incubators during the formation of the EE. Only after Shenzhen took off as ‘China’s Silicon Valley’ did local universities focus on entrepreneurship programmes. Attracted by Shenzhen’s entrepreneurship culture, top universities from other regions also established branches in Shenzhen. Hence, we propose that an EE is the result of intensive and collective entrepreneurship practices. These practices reinforce the entrepreneurship field and expand its boundaries, influencing various types of actors to engage in entrepreneurship.

Second, entrepreneurship patterns evolve over time. Our study applied Bourdieu’s theory, in an endeavour to explain the kinds of varied resources (capitals) that individuals draw on to enact their strategies and the negotiation and shaping of these strategies by a certain context (habitus). Hence, entrepreneurship capital and habitus bring out different entrepreneurship practice patterns depending on the EE’s development phase.

In the heteronomous phase of Shenzhen’s EE, the most valued form of capital was economic capital. During this period, entrepreneurs were inspired by the Reform and Opening-up policies; making money as much and as quickly was the dominant ethos in the area at that time. Meanwhile, with less institutional networks and events, the social capital that entrepreneurs could utilise was homogeneous; entrepreneurs often preferred or had to do business with family and friends rather than look for diverse partners or professional advisors. In this context, most entrepreneurs sensed and followed a habitus of making a quick profit. The practices of imitation or adaptation of global brand products were accepted and shared with families and close friends. Although they did not claim engaging in Schumpeterian entrepreneurship, they innovated the production process for speed and small-scale cost savings.

In the autonomous phase of the Shenzhen EE, entrepreneurs were more opportunity motivated and had a strong desire to promote indigenous innovation. Therefore, instead of economic capital, cultural capital played the most critical role in the entrepreneurship field. Increasingly, more entrepreneurs possessed postgraduate degrees from top-ranking universities overseas and were experienced in research and development. They took their identities as ‘entrepreneurs’ seriously and desired to be ‘tech-elite’. Hence, for the time being, instead of selling copycats, the entrepreneurship habitus changed to making enough profit through.

Third, over time, entrepreneurship competitions and related events changed entrepreneurship practices from distributed to integrated. This conclusion aligns with previous work studying Seattle’s EE (Thompson et al., 2018). Events such as science and technology exhibitions, entrepreneurship competitions, and other daily activities made connections and interactions between individuals more frequent and routine. As a result, the initially distributed entrepreneurs gradually developed into entrepreneur communities, creating coordinated and integrated entrepreneurship practices.

## Data availability statement

The original contributions presented in the study are included in the article/supplementary material, further inquiries can be directed to the corresponding author.

## Author contributions

MH provided research data, built the study framework and wrote the manuscript. ZG designed figures and provided scientific research funding support. CW refined the literature review part. All authors contributed to the article and approved the submitted version.

## Funding

The work is supported by the Yongjiang Social Science Fund for Early Career Researchers, the General Project of Social Science Research Fund of Ningbo (Grant No. JD-FZ140), the General Project of Philosophy and Social Science Planning Fund of Zhejiang Province (Grant No. 21NDJC170YB).

## Conflict of interest

The authors declare that the research was conducted in the absence of any commercial or financial relationships that could be construed as a potential conflict of interest.

## Publisher’s note

All claims expressed in this article are solely those of the authors and do not necessarily represent those of their affiliated organizations, or those of the publisher, the editors and the reviewers. Any product that may be evaluated in this article, or claim that may be made by its manufacturer, is not guaranteed or endorsed by the publisher.

## Abbreviations

CHTF: China Hi-Tech Fair
EE: Entrepreneurial Ecosystem
VC: Venture Capital

## References

[ref1] Acs Z. J. Stam E. Audretsch D. B. O’Connor A. (2017). The lineages of the entrepreneurial ecosystem approach. Small Bus. Econ. 49, 1–10. doi: 10.1007/s11187-017-9864-8

[ref2] Ahlstrom D. Bruton G. D. Yeh K. S. (2007). Venture capital in China: past, present, and future. Asia Pac. J. Manag. 24, 247–268. doi: 10.1007/s10490-006-9032-1

[ref4] Aoyama Y. (2009). Entrepreneurship and regional culture: the case of Hamamatsu and Kyoto, Japan. Reg. Stud. 43, 495–512. doi: 10.1080/00343400902777042

[ref5] Autio E. Nambisan S. Thomas L. D. W. Wright M. (2018). Digital affordances, spatial affordances, and the genesis of entrepreneurial ecosystems. Strateg. Entrep. J. 12, 72–95. doi: 10.1002/sej.1266

[ref6] Barba-Sánchez V. Mitre M. Del Brío J. (2022). The entrepreneurial intention of university students: an environmental perspective. Eur. Res. Manag. Bus. Econ. 28:100184. doi: 10.1016/j.iedeen.2021.100184

[ref7] Bathelt H. Glückler J. (2014). Institutional change in economic geography. Prog. Hum. Geogr. 38, 340–363. doi: 10.1177/0309132513507823

[ref9] Bourdieu P. (1984). Distinction: A Social Critique of the Judgement of Taste. London: Routledge & Kegan Paul.

[ref10] Bourdieu P. (1990). The Logic of Practice. Palo Alto, CA: Stanford University Press.

[ref11] Brown R. Mason C. (2017). Looking inside the spiky bits: a critical review and conceptualisation of entrepreneurial ecosystems (journal article). Small Bus. Econ. 49, 11–30. doi: 10.1007/s11187-017-9865-7

[ref12] Bruns K. Bosma N. Sanders M. Schramm M. (2017). Searching for the existence of entrepreneurial ecosystems: a regional cross-section growth regression approach (journal article). Small Bus. Econ. 49, 31–54. doi: 10.1007/s11187-017-9866-6

[ref13] Cansiz M. Tekneci P. D. (2018). Innovative and technology-based women entrepreneurs in Turkey: capital and performance. J. Econ. Cult Soc. 57, 151–183. doi: 10.26650/JECS372449

[ref14] Cederberg M. Villares-Varela M. (2019). Ethnic entrepreneurship and the question of agency: the role of different forms of capital, and the relevance of social class. J. Ethn. Migr. Stud. 45, 115–132. doi: 10.1080/1369183X.2018.1459521

[ref16] Civera A. Meoli M. Vismara S. (2019). Do academic spinoffs internationalise? J. Technol. Transfer. 44, 381–403. doi: 10.1007/s10961-018-9683-3

[ref17] Cohen B. (2006). Sustainable valley entrepreneurial ecosystems. Bus. Strateg. Environ. 15, 1–14. doi: 10.1002/bse.428

[ref18] Cooke P. (2007). Regional innovation, entrepreneurship and talent systems. Int. J. Entrep. Innov. Manag. 7, 117–139. doi: 10.1504/IJEIM.2007.012878

[ref19] Cukier D. Kon F. Krueger N. (2015). Designing a maturity model for software startup ecosystems. Lect. Notes Comput. Sci 9459. doi: 10.1007/978-3-319-26844-6_45

[ref20] Davidsson P. (1995). Culture, structure and regional levels of entrepreneurship. Entrep. Reg. Dev. 7, 41–62. doi: 10.1080/08985629500000003

[ref22] Dodd S. D. Pret T. Shaw E. (2016). “Advancing understanding of entrepreneurial embeddedness: Forms of capital, social contexts and time” in A Research Agenda for Entrepreneurship and Context. eds. Welter F. Gartner W. B. (Cheltenham: Edward Elgar Publishing)

[ref24] Elston J. A. Weidinger A. (2019). Entrepreneurial intention and regional internationalisation in China. Small Bus. Econ. 53, 1001–1015. doi: 10.1007/s11187-018-0114-5

[ref27] Forson C. Özbilgin M. Ozturk M. B. Tatli A. (2014). “Multi-level approaches to entrepreneurship and small business research-transcending dichotomies with Bourdieu” in Handbook of Research on Small Business and Entrepreneurship. eds. Welter F. Gartner W. B. (Cheltenham: Edward Elgar Publishing)

[ref1004] Feldman M. P. (2001). The entrepreneurial event revisited: firm formation in a regional context. Ind. Corp. Chang. 10, 861–891.

[ref28] Garud R. Gehman J. Giuliani A. P. (2014). Contextualising entrepreneurial innovation: a narrative perspective. Res. Policy 43, 1177–1188. doi: 10.1016/j.respol.2014.04.015

[ref1003] Granovetter M. S. (1973). The strength of weak ties. Amer. J. Sociol. 78, 1360–1380.

[ref30] Griffin-El E. Olabisi J. (2017). “Homegrown: the role of cultural origins in the bricolage activity of immigrant entrepreneurship” in Academy of Management Proceedings. eds. Welter F. Gartner W. B. (Briarcliff Manor, NY: Academy of Management), 14780.

[ref1002] Hsu D. H. Kenney M. (2005). Organizing venture capital: the rise and demise of American Research & Development Corporation, 1946–1973. Ind. Corp. Chang. 14, 579–616.

[ref1001] Feld B. (2012). Startup Communities: Building an Entrepreneurial Ecosystem in Your City[M]. Hoboken, New Jersey: John Wiley & Sons.

[ref32] Isenberg D. (2011). The entrepreneurship ecosystem strategy as a new paradigm for economic policy: principles for cultivating entrepreneurship. Presentation at the Institute of International and European Affairs, pp. 1–13.

[ref33] Keane M. Zhao E. J. (2012). Renegades on the frontier of innovation: the Shanzhai grassroots communities of Shenzhen in China’s creative economy. Eurasian Geogr. Econ. 53, 216–230. doi: 10.2747/1539-7216.53.2.216

[ref34] Knox S. Arshed N. (2021). Network governance and coordination of a regional entrepreneurial ecosystem. Reg. Stud. 56, 1161–1175. doi: 10.1080/00343404.2021.1988067

[ref35] Laspita S. Sitaridis I. Kitsios F. Sarri K. (2023). Founder or employee? The effect of social factors and the role of entrepreneurship education. J. Bus. Res. 155:113422. doi: 10.1016/j.jbusres.2022.113422

[ref36] Lee R. Shaw E. (2016). Bourdieu’s non-material forms of capital: implications for start-up policy. Environ. Plann. C 34, 1734–1758. doi: 10.1177/0263774X16638850

[ref37] Leitch C. M. McMullan C. Harrison R. T. (2013). The development of entrepreneurial leadership: the role of human, social and institutional capital. Br. J. Manag. 24, 347–366. doi: 10.1111/j.1467-8551.2011.00808.x

[ref39] Malecki E. J. (2018). Entrepreneurship and entrepreneurial ecosystems. Geogr. Compass. 12:e12359. doi: 10.1111/gec3.12359

[ref40] Marshall A. (1920). Principals of Economics. Macmillian: London

[ref41] Maton K. (2005). A question of autonomy: Bourdieu’s field approach and higher education policy. J. Educ. Policy 20, 687–704. doi: 10.1080/02680930500238861

[ref42] McAdam M. Harrison R. T. Leitch C. M. (2019). Stories from the field: women’s networking as gender capital in entrepreneurial ecosystems. Small Bus. Econ. 53, 459–474. doi: 10.1007/s11187-018-9995-6

[ref43] Mohammadi N. Karimi A. (2021). Entrepreneurial ecosystem big picture: a bibliometric analysis and co-citation clustering. J. Res. Mark. Entrep. 24, 23–38. doi: 10.1108/JRME-10-2020-0141

[ref44] Molina-Ramírez E. Barba-Sánchez V. (2021). Embeddedness as a differentiating element of indigenous entrepreneurship: insights from Mexico. Sustainability 13:2117. doi: 10.3390/su13042117

[ref46] Nambisan S. (2018). Architecture vs. ecosystem perspectives: reflections on digital innovation. Inf. Organ. 28, 104–106. doi: 10.1016/j.infoandorg.2018.04.003

[ref47] Navarro Z. (2006). In search of a cultural interpretation of power: the contribution of Pierre Bourdieu. IDS Bull. 37, 11–22. doi: 10.1111/j.1759-5436.2006.tb00319.x

[ref48] Neumeyer X. Santos S. C. Caetano A. Kalbfleisch P. (2019). Entrepreneurship ecosystems and women entrepreneurs: a social capital and network approach. Small Bus. Econ. 53, 475–489. doi: 10.1007/s11187-018-9996-5

[ref49] Opute A. P. Kalu K. I. Adeola O. Iwu C. G. (2021). Steering sustainable economic growth: entrepreneurial ecosystem approach. J. Entrep. Innov. Emerg. Econ. 7, 216–245. doi: 10.1177/23939575211024384

[ref50] Ozcan P. Han S. Graebner M. E. (2017). “Single cases: the what, why, and how” in The Routledge Companion to Qualitative Research in Organisation Studies. eds. Mir R. A. A. J. Sanjay J. (New York, NY: Routledge), 92–112.

[ref51] Packard M. D. Bylund P. L. (2018). On the relationship between inequality and entrepreneurship. Strateg. Entrep. J. 12, 3–22. doi: 10.1002/sej.1270

[ref52] Porter M. (1980). Competitive Strategy. New York: Free Press.

[ref53] Pugh R. Soetanto D. Jack S. L. Hamilton E. (2021). Developing local entrepreneurial ecosystems through integrated learning initiatives: the Lancaster case. Small Bus. Econ. 56, 833–847. doi: 10.1007/s11187-019-00271-5

[ref54] Radinger-Peer V. Sedlacek S. Goldstein H. (2018). The path-dependent evolution of the entrepreneurial ecosystem (ee)—dynamics and region-specific assets of the case of Vienna (Austria). Eur. Plan. Stud. 26, 1499–1518. doi: 10.1080/09654313.2018.1494136

[ref55] Rippa P. Ferruzzi G. Holienka M. Capaldo G. Coduras A. (2020). What drives university engineering students to become entrepreneurs? Finding different recipes using a configuration approach. J. Small Bus. Manag. 1-31, 1–31. doi: 10.1080/00472778.2020.1790291

[ref56] Roundy P. T. Brockman B. K. Bradshaw M. (2017). The resilience of entrepreneurial ecosystems. J. Bus. Ventur. Insights 8, 99–104. doi: 10.1016/j.jbvi.2017.08.002

[ref57] Roundy P. T. Lyons T. S. (2022). Where are the entrepreneurs? A call to theorise the micro-foundations and strategic organisation of entrepreneurial ecosystems. Strateg. Organ. doi: 10.1177/14761270211056240,147612702110562

[ref58] Saxenian A. (1996). Regional Advantage. Cambridge, MA: Harvard University Press.

[ref59] Sklaveniti C. Steyaert C. (2020). Reflecting with Pierre Bourdieu: towards a reflexive outlook for practice-based studies of entrepreneurship. Entrep. Reg. Dev. 32, 313–333. doi: 10.1080/08985626.2019.1641976

[ref60] Sorenson O. (2018). Social networks and the geography of entrepreneurship. Small Bus. Econ. 51, 527–537. doi: 10.1007/s11187-018-0076-7

[ref61] Spigel B. (2017). The relational organization of entrepreneurial ecosystems. Entrep. Theory Pract. 41, 49–72. doi: 10.1111/etap.12167

[ref62] Spigel B. Harrison R. (2018). Toward a process theory of entrepreneurial ecosystems. Strateg. Entrep. J. 12, 151–168. doi: 10.1002/sej.1268

[ref63] Stam E. (2015). Entrepreneurial ecosystems and regional policy: a sympathetic critique. Eur. Plan. Stud. 23, 1759–1769. doi: 10.1080/09654313.2015.1061484

[ref64] Stam E. Spigel B. (2016). Entrepreneurial Ecosystems, U.S.E. Discussion paper series.

[ref65] Storper M. Salais R. (1997). Worlds of Production: The Action Frameworks of the Economy. Cambridge, MA: Harvard University Press.

[ref66] Tatli A. Vassilopoulou J. Özbilgin M. Forson C. Slutskaya N. (2014). A Bourdieuan relational perspective for entrepreneurship research. J. Small Bus. Manag. 52, 615–632. doi: 10.1111/jsbm.12122

[ref67] Thompson T. A. Purdy J. M. Ventresca M. J. (2018). How entrepreneurial ecosystems take form: evidence from social impact initiatives in Seattle. Strateg. Entrep. J. 12, 96–116. doi: 10.1002/sej.1285

[ref68] Turkina E. (2018). The importance of networking to entrepreneurship: Montreal’s artificial intelligence cluster and its born-global firm element AI. J. Small Bus. Entrep. 30, 1–8. doi: 10.1080/08276331.2017.1402154

[ref70] Vershinina N. Barrett R. Meyer M. (2011). Forms of capital, intra-ethnic variation and polish entrepreneurs in Leicester. Work Employ. Soc. 25, 101–117. doi: 10.1177/0950017010389241

[ref71] Villanueva C. E. Angeles A. Revilla L. C. (2018). Tying strong ties in informal entrepreneurship: a constraint or an entrepreneurial driver? J. Dev. Entrep. 23:1850002. doi: 10.1142/S1084946718500024

[ref72] Wei Y. (2022). Regional governments and opportunity entrepreneurship in underdeveloped institutional environments: an entrepreneurial ecosystem perspective. Res. Policy 51:104380. doi: 10.1016/j.respol.2021.104380

[ref73] White S. Gao J. Zhang W. (2005). Financing new ventures in China: system antecedents and institutionalisation. Res. Policy 34, 894–913. doi: 10.1016/j.respol.2005.04.002

[ref74] Yamamura S. Lassalle P. (2020). Proximities and the emergence of regional industry: evidence of the liability of smallness in Malta. Eur. Plan. Stud. 28, 380–399. doi: 10.1080/09654313.2019.1668915

[ref75] York J. G. Venkataraman S. (2010). The entrepreneur–environment nexus: uncertainty, innovation, and allocation. J. Bus. Ventur. 25, 449–463. doi: 10.1016/j.jbusvent.2009.07.007

[ref76] Zhou T. (2022). Understanding users’ contribution in open innovation communities: a social capital perspective. Kybernetes 51, 937–951. doi: 10.1108/K-10-2020-0665

